# Fate Determination of ZnO in Commercial Foods and Human Intestinal Cells

**DOI:** 10.3390/ijms21020433

**Published:** 2020-01-09

**Authors:** Ye-Rin Jeon, Jin Yu, Soo-Jin Choi

**Affiliations:** Division of Applied Food System, Major of Food Science & Technology, Seoul Women’s University, Seoul 01797, Korea; yrjeon0715@swu.ac.kr (Y.-R.J.); ky5031@swu.ac.kr (J.Y.)

**Keywords:** zinc oxide, fate, cloud point extraction, dissolution, commercial food, intestinal absorption

## Abstract

(1) Background: Zinc oxide (ZnO) particles are widely used as zinc (Zn) fortifiers, because Zn is essential for various cellular functions. Nanotechnology developments may lead to production of nano-sized ZnO, although nanoparticles (NPs) are not intended to be used as food additives. Current regulations do not specify the size distribution of NPs. Moreover, ZnO is easily dissolved into Zn ions under acidic conditions. However, the fate of ZnO in commercial foods or during intestinal transit is still poorly understood. (2) Methods: We established surfactant-based cloud point extraction (CPE) for ZnO NP detection as intact particle forms using pristine ZnO-NP-spiked powdered or liquid foods. The fate determination and dissolution characterization of ZnO were carried out in commercial foods and human intestinal cells using in vitro intestinal transport and ex vivo small intestine absorption models. (3) Results: The results demonstrated that the CPE can effectively separate ZnO particles and Zn ions in food matrices and cells. The major fate of ZnO in powdered foods was in particle form, in contrast to its ionic fate in liquid beverages. The fate of ZnO was closely related to the extent of its dissolution in food or biomatrices. ZnO NPs were internalized into cells in both particle and ion form, but dissolved into ions with time, probably forming a Zn–ligand complex. ZnO was transported through intestinal barriers and absorbed in the small intestine primarily as Zn ions, but a small amount of ZnO was absorbed as particles. (4) Conclusion: The fate of ZnO is highly dependent on food matrix type, showing particle and ionic fates in powdered foods and liquid beverages, respectively. The major intracellular and intestinal absorption fates of ZnO NPs were Zn ions, but a small portion of ZnO particle fate was also observed after intestinal transit. These findings suggest that the toxicity of ZnO is mainly related to the Zn ion, but potential toxicity resulting from ZnO particles cannot be completely excluded.

## 1. Introduction

Nanomaterials have been widely applied to diverse industries, such as electronics, medicine, pharmaceutics, cosmetics, and foods. In particular, food additive particles, including silicon dioxide (SiO_2_), titanium dioxide (TiO_2_), and zinc oxide (ZnO) are widely used as anticaking agents, coloring agents, and nutritional fortifiers, respectively [1,2,3]. Among them, ZnO can be added to milk, dairy products, cereals, and beverages. This is due to the fact that zinc (Zn) plays an essential role as a trace element in the immune system, cell function, enzyme activity, and signaling in human body [4,5,6]. ZnO is used as a food additive in the United States (US) and Europe, and classified as “Generally Recognized as Safe” by the US Food and Drug Administration [7]. The acceptable daily intake (ADI) for Zn in Zn-fortified functional foods is in the range of 2.55 to 12 mg in the Republic of Korea [8]. Nanotechnology developments have led to the production of nano-sized ZnO particles, although nanomaterials that range in size from 1 to 100 nm are not intended to be used as food additives. Indeed, the particle size distribution for ZnO as a food additive is unspecified [9].

The fate determination of ZnO is of importance for interpreting and understanding its potential toxicity, because the toxic effects resulting from ZnO nanoparticles (NPs), NP aggregates/agglomerates, micro-sized particles, or Zn ions are completely different [10,11]. A few reports have demonstrated the fates of ZnO in biological systems or environmental water samples [12,13,14]. ZnO NPs were reported to be primarily present as ionic forms in tissues after oral administration in rats [15,16,17]. Zn ion release from ZnO NPs in simulated gastric fluid increased to 14% has also been reported [18,19], suggesting their partial dissolution property. Studies have demonstrated that ZnO can be easily decomposed to Zn ions in acidic solutions or biological fluids [20,21,22,23]. Since most foods have a slightly acidic pH and Zn-fortified foods are taken up by the oral route, ZnO can be dissolved into Zn ions in food matrices to some extent, and does not present in its particle form in the body. However, there is no clear information about the fate of ZnO in commercial foods and human intestinal cells during intestinal transit.

Food additive ZnO is directly added to complex food matrices in solution or powdered form. Hence, the interactions between ZnO and food components should also be considered. Indeed, ZnO NPs have been demonstrated to interact with food components, such as proteins and saccharides, forming particle–matrix corona (nanocorona covered by matrices) [23,24,25]. These interactions were also found to affect cytotoxicity, intestinal transport efficiency, and oral absorption [24,25,26,27]. Thus, separating ZnO from complex food matrices in intact particle form is challenging. Currently, the most widely applied method for detecting ZnO is inductively coupled plasma–atomic emission spectroscopy (ICP-AES), ICP–mass spectroscopy, or atomic absorption spectroscopy. However, these methods require pre-digestion procedures with acids to digest organic matrices, and therefore are used for analyzing total Zn levels only and cannot be used to distinguish ZnO particles from Zn ions.

On the other hand, cloud point extraction (CPE) using Triton X-114 (TX-114) was first described and developed for the analysis of trace NPs, such as gold, silver, and copper as particle forms [28,29,30]. TX-114 is a surfactant, is cost-effective, and has the advantage of forming micelles at room temperature (20–25 °C), contributing to easy manipulation. To date, TX-114-based CPE studies have focused on the determination of silver NPs in water or cells [31,32]. ZnO separation by CPE in aqueous phase or environmental waters where little organic matrix is present has also been reported [33]. A CPE-based approach was used for the detection of aluminum (Al) or Zn in foodstuffs. However, these studies used ethylenediaminetetraacetic acid (EDTA) or 8-hydroxyquinoline as chelating agents for precipitation of Al or Zn ions in precipitated surfactant-rich phase by CPE [34,35]. Moreover, the CPE method was applied after pre-treatments such as filtering, nitric acid treatment, and dry-ashing were applied in order to digest organic matrices in foods [34,35]. No attempt has been made to detect ZnO in its particle form in complex systems, such as in foods or biomatrices.

In the present study, an analytical method for determining ZnO with intact particle size and shape in foods and human intestinal cells was established using surfactant-based CPE. The fates of ZnO in commercial processed foods, including liquid and powdered types, were determined based on the optimized method established with pristine ZnO-NP-spiked food matrices. Finally, fate determination was assessed in human intestinal cells, in vitro models of human intestinal barriers, and an ex vivo intestinal absorption model.

## 2. Results

### 2.1. Optimization and Characterization of CPE for ZnO NPs

**Figure 1:**
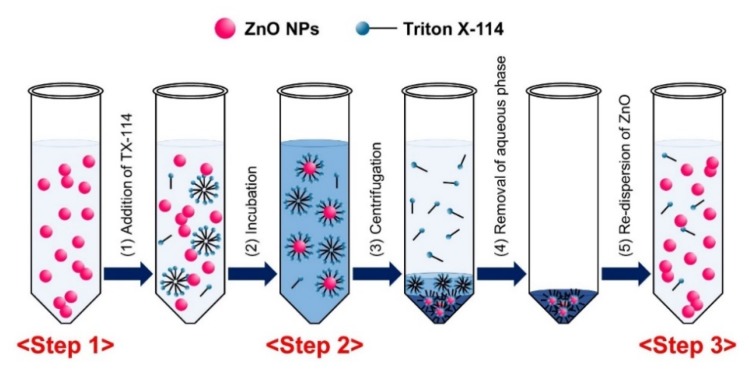
Schematic illustration of Triton X-114 (TX-114)-based cloud point extraction (CPE) procedure.

**Figure 2:**
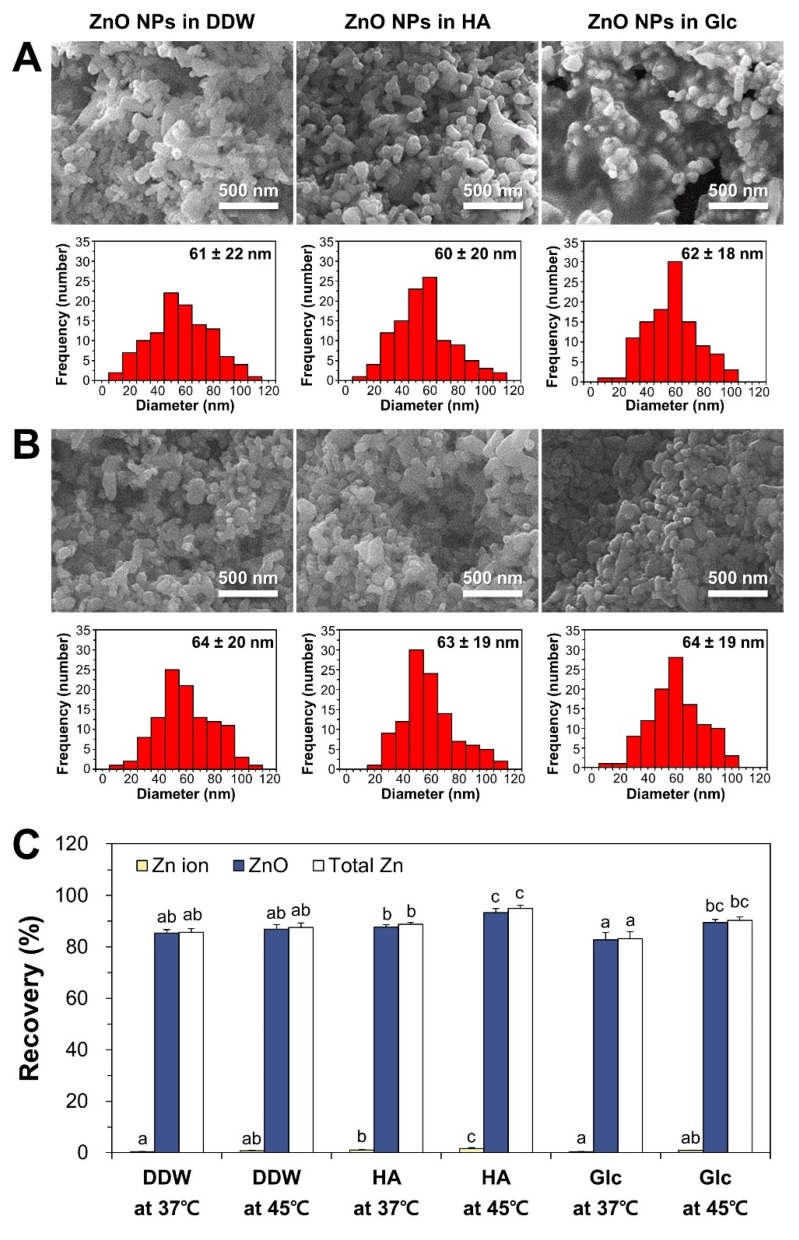
Field emission-scanning electron microscopy (FE-SEM) images and size distribution of pristine ZnO NPs in different dispersants (**A**) before CPE and (**B**) after CPE. (**C**) Recovery (%) of Zn ions, ZnO particles, and total Zn levels obtained from pristine ZnO NPs by CPE. Size distributions were obtained by randomly selecting 100 particles from FE-SEM images. Different lower-case letters (a,b,c) indicate significant differences among different CPE conditions (*p* < 0.05).

TX-114-based CPE was first optimized utilizing pristine ZnO NPs. The optimization of ZnO dispersant was carried out with humic acid (HA) and glucose (Glc), and compared with NPs in distilled and deionized water (DDW) as a control at 37 and 45 °C, respectively. HA was chosen as a dispersing agent based on the research of Majedi et al. [33], and Glc is known to play a role in NP dispersion as well [25,36]. The physicochemical properties of ZnO NPs after dispersion (Step 1), during CPE (Step 2: surfactant-rich and aqueous phases without centrifugation), and after CPE (Step 3: surfactant-rich and aqueous phases separated by centrifugation) were characterized (Figure 1). When particle size, shape, and morphology were examined by field emission-scanning electron microscopy (FE-SEM), pristine ZnO NPs dispersed in DDW, HA, and Glc (Step 1) had an average primary particle size of ~60 nm with irregular shapes (Figure 2A). The precipitated, TX-114-rich phase of ZnO NPs after CPE (Step 3) had similar primary particle size distributions compared to pristine NPs in different dispersants, showing no significant differences in particle size between Step 1 and Step 3 (Figure 2B, *p* > 0.05). FE-SEM images during CPE process (Step 2) could not be clearly obtained due to the presence of high level of organic material TX-114.

The hydrodynamic radii of ZnO NPs consequently increased when ZnO NPs were covered by TX-114 and after micelle formations (Step 2). Thus, the formation of ZnO NPs-TX-114 micelles was confirmed through dynamic light scattering (DLS) analysis. The hydrodynamic diameters of pristine ZnO NPs in DDW or HA were significantly smaller than those in Glc at Step 1 (Table 1). The hydrodynamic radii of ZnO NPs in different dispersants dramatically increased during the CPE process (Step 2) in all cases, but decreased to the same levels similar to that in pristine NPs after CPE (Step 3), only when ZnO NPs were dispersed in HA (Table 1). No statistical differences in temperatures (37 and 45 °C) were found (*p* > 0.05), except in ZnO NPs in HA during CPE (Step 2). On the other hand, the zeta potential values changed to slightly negative charges in all cases after CPE (Step 3, Table 1).

### 2.2. Recovery of Pristine ZnO NPs by CPE

The recovery (%) of ZnO NPs in different dispersants by CPE was checked by quantifying Zn amounts in both aqueous and TX-114-rich phases (supernatant and precipitate after CPE, respectively) in order to confirm the efficiency of the CPE procedure. As shown in Figure 2C, more than 82% of the ZnO NPs were recovered from the precipitates as particles after CPE, regardless of CPE conditions. Only small portions (less than 2%) of ZnO NPs were detected in supernatants as ions after CPE. The highest total recovery (94.9%) of ZnO NPs in both supernatant and precipitate after CPE was found when ZnO NPs were dispersed in HA at 45 °C. It is worth noting that 93.4% of pristine ZnO NPs in HA were obtained in the precipitates as particles after CPE. Hence, this condition was further used for fate determination of ZnO in commercial foods and human intestinal cells.

### 2.3. Dissolution Property of ZnO NPs in Food/Bio Matrices

**Figure 3:**
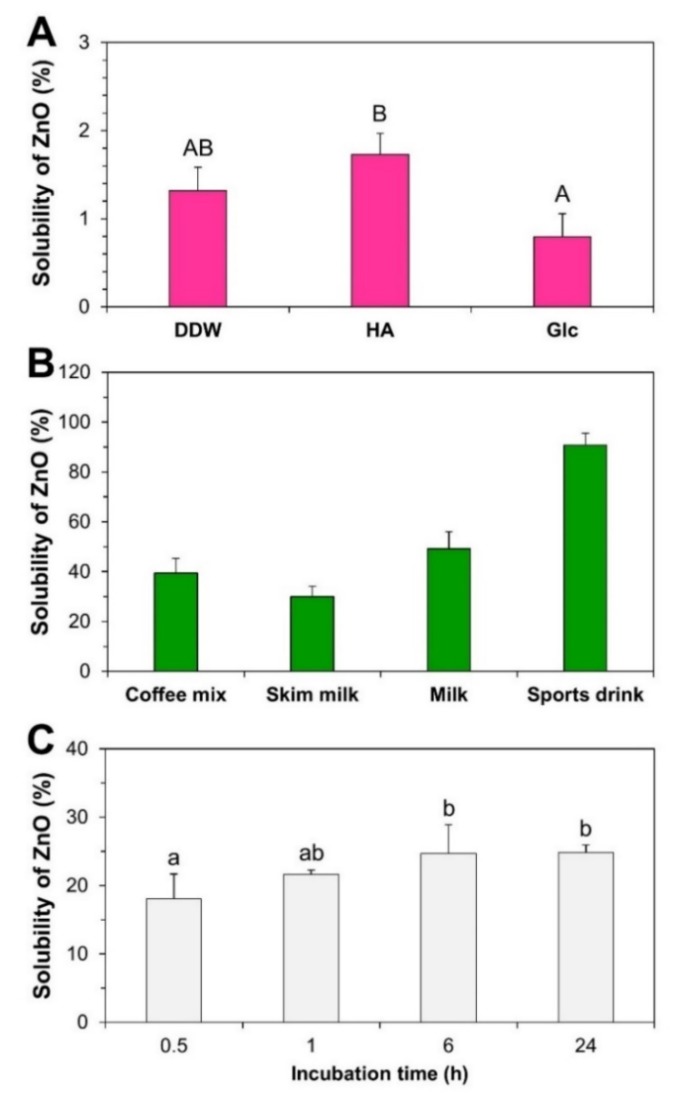
Dissolution properties of ZnO NPs in (**A**) dispersants, (**B**) food matrices, and (**C**) cell culture medium. Different upper-case letters (A,B) indicate significant differences among different dispersants (*p* < 0.05). Different lower-case letters (a,b) indicate significant differences among incubation times (*p* < 0.05).

The solubility of ZnO NPs in food matrices and cell culture medium was first checked prior to fate determination. Two different food types which were representative of powdered or liquid foods were used. Powdered foods (coffee mix and skim milk) and liquid beverages (milk and sports drink) were selected based on the potential utilization of ZnO in Zn-fortified foods. Figure 3A showed that the solubilities of ZnO NPs in different dispersants ranged from 0.8% to 1.7%. The pHs of ZnO dispersed in DDW, HA, and Glc were 8.6, 8.1, and 8.7, respectively.

On the other hand, the solubilities of ZnO in food matrices increased, reaching 39.4%, 30.0%, and 49.2% in coffee mix, skim milk, and milk, respectively. There was a dramatic increase in solubility observed in sports drink, which reached up to 90.9% (Figure 3B). The pHs of ZnO-spiked food matrices were 6.2, 6.9, 6.9, and 3.3 in coffee mix, skim milk, milk, and sports drink, respectively. The solubility of ZnO in cell culture minimum essential medium (MEM) (pH 7.0) was ~18% after 0.5 h and increased up to 24.8% after 6–24 h (Figure 3C).

### 2.4. Characterization and Fate of ZnO-NP-Spiked Foods

**Figure 4:**
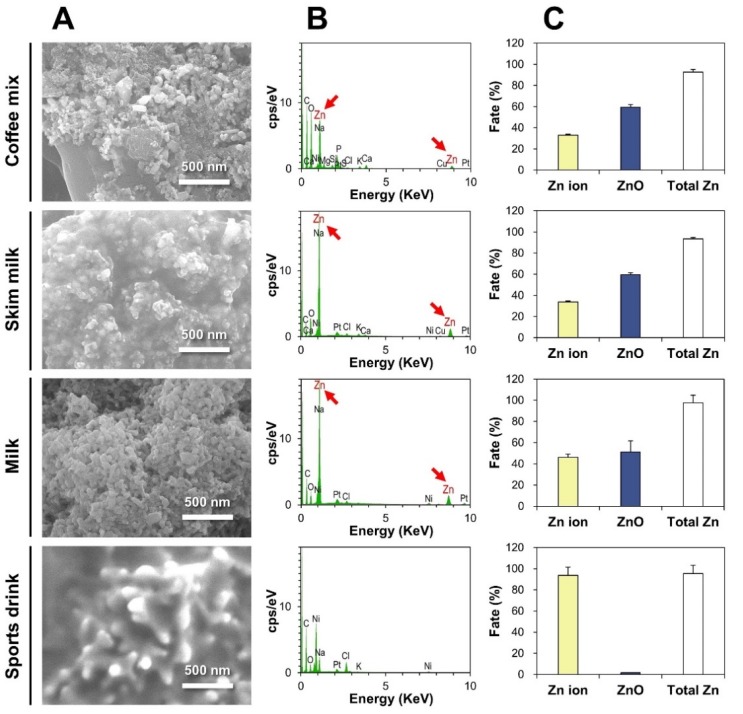
(**A**) FE-SEM images and (**B**) energy dispersive X-ray spectroscopy (EDS) analysis of the precipitates of ZnO-NP-spiked foods after CPE. (**C**) Fate (%) of ZnO NPs in ZnO-NP-spiked foods by CPE, followed by inductively coupled plasma–atomic emission spectroscopy (ICP-AES) quantification.

The reliability and accuracy of the CPE method for fate determination of ZnO in commercial foods were checked using ZnO-NP-spiked foods. DLS results demonstrated that all ZnO NPs recovered from the precipitates of ZnO-NP-spiked coffee mix, skim milk, and milk after CPE had statistically significant similarities in hydrodynamic radii compared to pristine ZnO NPs (*p* > 0.05, Figure S1). On the other hand, no particle forms were detected in the precipitates of ZnO-NP-spiked sports drink after CPE (Figure S1). SEM and energy dispersive X-ray spectroscopy (EDS) analysis revealed the presence of Zn elements recovered from the precipitated TX-114-rich phases of all ZnO-NP-spiked foods after CPE, except sports drink (Figure 4A,B).

Quantitative ICP-AES analysis results on the supernatants and precipitates after CPE are presented in Figure 4C. About 59.5%, 59.5%, and 51.3% of ZnO were present as particle forms in coffee mix, skim milk, and milk, respectively, and 93.7% of ZnO NPs were dissolved and detected as Zn ions in sports drink. The total Zn recoveries (%) from both Zn ions and ZnO particles ranged from 92.6% to 97.5%.

### 2.5. Fate of ZnO in Commercial Foods

**Figure 5:**
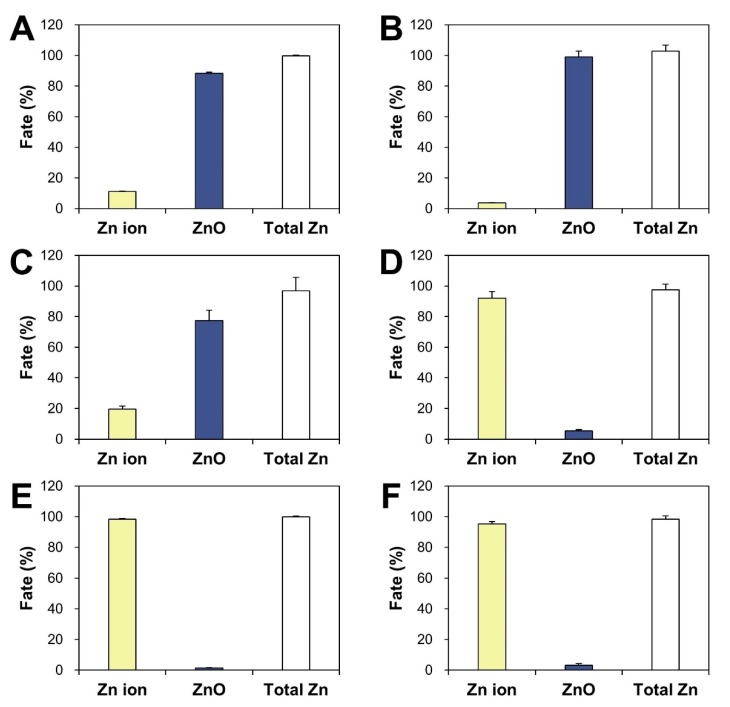
Fate (%) of ZnO in ZnO-added commercial foods: (**A**) chocolate powder, (**B**) powdered probiotics, (**C**) cereals, (**D**) functional peptide beverage, (**E**) fruit juice. (**F**) Fate of Zn gluconate in a functional mineral beverage by CPE.

The presence of ZnO particles or Zn ions in Zn-fortified commercial foods was determined based on the established CPE method. Commercial foods that indicated ZnO as an additive as seen in product labeling were chosen, and another product containing Zn gluconate as a Zn fortifier was also used for comparative study. When the supernatants and precipitates after CPE were quantitatively analyzed by ICP-AES, the fate of ZnO differed depending on food type (Figure 5). The highest concentration of ZnO was detected as particle forms (~77–99%) in powdered or dried foods (chocolate powder, powdered probiotics, and cereals). ZnO was determined to be mainly present as Zn ions (~92–98%) in liquid beverages (functional peptide beverage and fruit juice). It is worth noting that the Zn ionic fate of Zn gluconate after CPE was also found in another functional mineral beverage (Figure 5F). The pHs of peptide, fruit juice, and mineral beverages were 4.7, 3.4, and 2.7, respectively, whereas the pHs of powdered chocolate, probiotics, and cereals were 7.3, 7.5, and 6.5, respectively. The total Zn recoveries (%) from both ZnO particles and Zn ions ranged from 96.9% to 102.8%, which was calculated based on composition labeling in commercial foods.

### 2.6. Intracellular Fate of ZnO NPs

**Figure 6:**
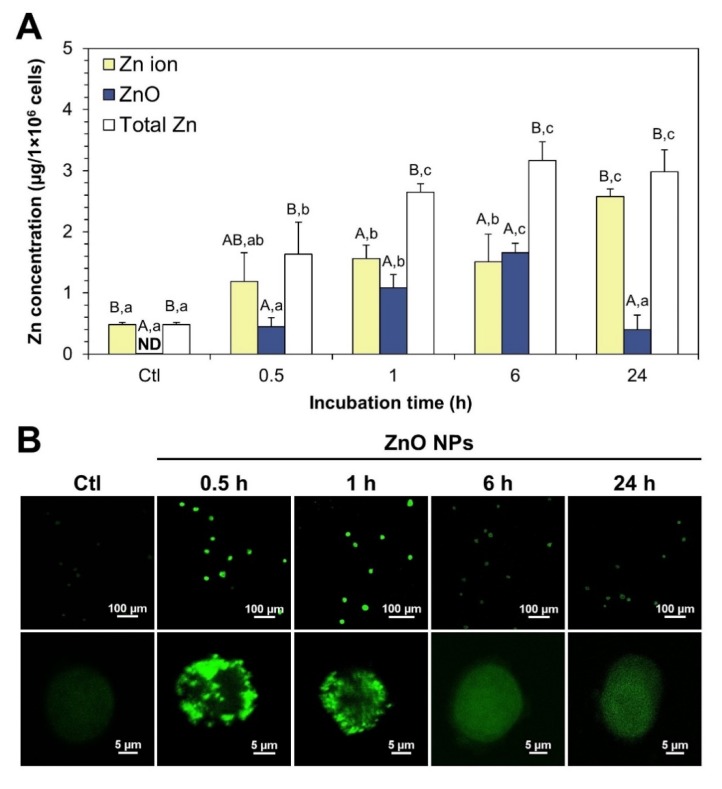
(**A**) Intracellular fate of ZnO NPs in human intestinal Caco-2 cells, as determined by CPE followed by ICP-AES analysis. (**B**) Confocal microscopic images of intracellular Zn ions, as determined by a Zn-selective fluorescent probe, TSQ in Caco-2 cells. Different upper-case letters (A,B) indicate significant differences among Zn ion, ZnO, and total Zn (*p* < 0.05). Different lower-case letters (a,b,c) indicate significant differences among different incubation times (*p* < 0.05). Abbreviation: ND, not detectable.

The fate of ZnO NPs was evaluated by CPE in human intestinal cells, and intracellular Zn levels in both supernatants and precipitates after CPE were quantified after cell lysis. Figure 6A shows that the uptake of ZnO NPs, as measured by total Zn levels, increased in concert with incubation time and reached a plateau at 1 h post-incubation. Intracellular ZnO NPs separated by CPE increased with time and reached a maximum level at 6 h, and then returned to normal control level after 24 h. On the other hand, intracellular Zn ion concentrations gradually increased and the highest Zn ion level was detected at 24 h, which was statistically similar to total Zn level at 24 h. When we compared ZnO NP/Zn ion ratios, most ZnO NPs were present as Zn ions (~71%) at 0.5 h, and both ZnO NPs and Zn ions were present at almost similar levels at 6 h (~53 vs. 47%). Zn ion forms (~87%) were primarily found at 24 h.

The intracellular fate of ZnO NPs was also checked with a Zn-selective fluorescent probe during incubation and examined by confocal microscopy. Figure 6B shows that the fluorescence intensity increased at 0.5–1 h and decreased thereafter. Magnified confocal images clearly demonstrated that the fluorescence intensity inside cells resulting from Zn ions was higher at 0.5–1 h than that at 6–24 h. Slightly increased fluorescence was also observed in control cells without ZnO NPs, attributed to basal intracellular Zn ion levels.

### 2.7. Intestinal Transport and Absorption Fate of ZnO NPs

**Figure 7:**
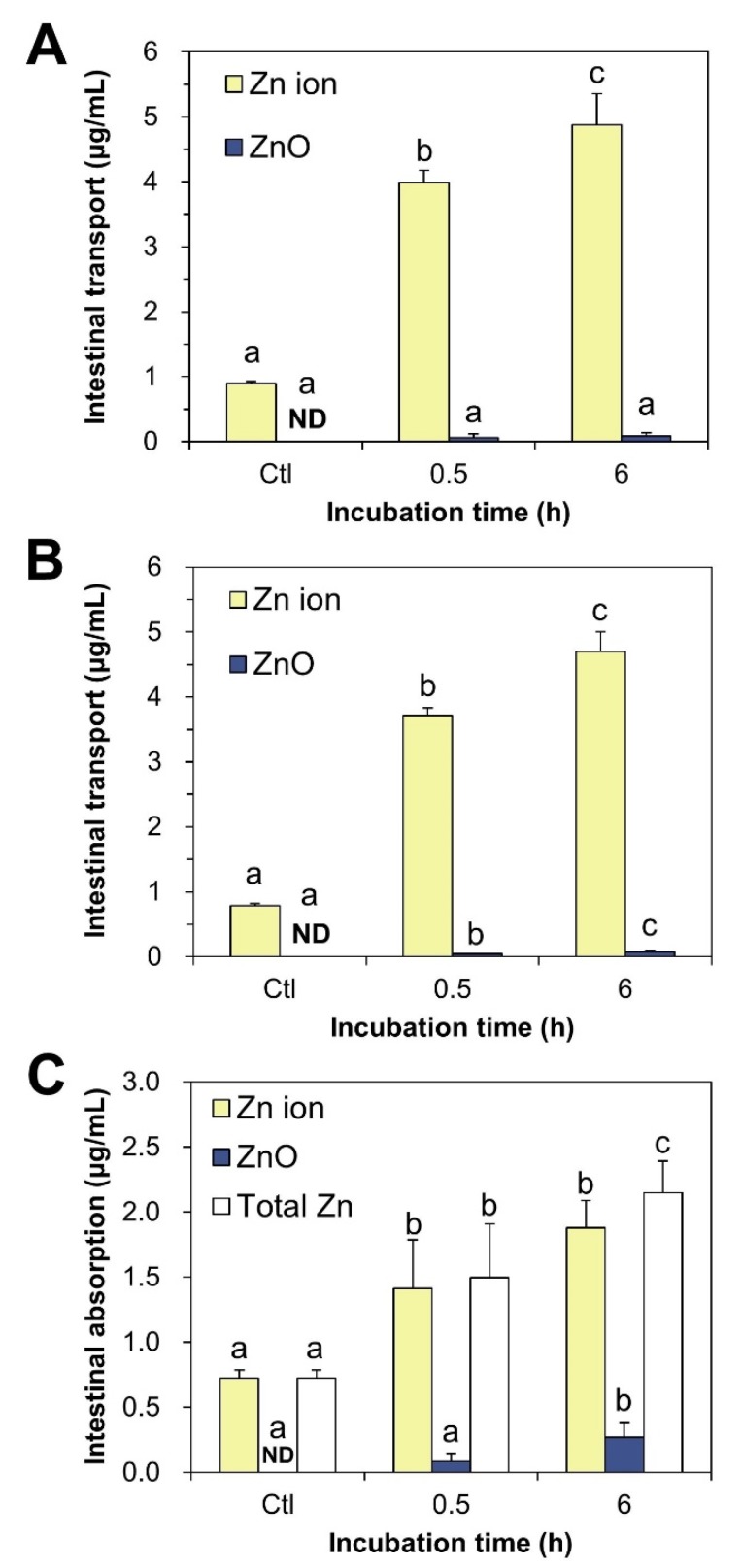
Intestinal transport fate of ZnO NPs obtained from in vitro models of (**A**) Caco-2 monolayer and (**B**) follicle-associated epithelium (FAE) by CPE. (**C**) Intestinal absorption fate of ZnO NPs using ex vivo everted small intestine sacs. Different lower-case letters (a,b,c) indicate significant differences among different incubation times (*p* < 0.05). Abbreviation: ND, not detectable.

The fate of ZnO NPs after intestinal transport was examined using in vitro Caco-2 monolayer and follicle-associated epithelium (FAE) models, representing the intestinal tight junction and microfold (M) cells in Peyer’s patches, respectively. Figure 7A,B show that ZnO NPs were transported through both Caco-2 monolayer and M cells primarily in Zn ion form. No significant differences in transport amount were found between the Caco-2 monolayer and M cells (*p* > 0.05).

We also evaluated the intestinal absorption fate of ZnO NPs using ex vivo everted small intestine sacs, which reflect small intestinal absorption in vivo. These results demonstrated that ZnO NPs were primarily absorbed into the body across the mucosa of the small intestine as Zn ionic forms (Figure 7C). It is also worth noting that ZnO can be slightly but significantly transported by M cells and absorbed in the small intestine in particle form after 0.5–6 h (Figure 7B,C).

## 3. Discussion

ZnO NPs have a wide range of applications in products intended for human consumption, such as foods and cosmetics, which raises increasing concerns about their potential toxicity. The fate determination of NPs is important, and can answer the fundamental question of whether NPs are present as intact particles or dissolved ionized forms. This type of research is also useful for understanding whether the toxicity of ZnO results from nano-sized particles or from Zn ions. In particular, fate determination is crucial for partially soluble NPs in commercial products or biological environments, such as ZnO NPs and silver NPs [37,38]. However, separating NPs and ionized forms from complex food and biological matrices without affecting intact particle size and morphology is challenging.

In this study, TX-114-based CPE was first optimized using pristine ZnO NPs. The results obtained revealed that ZnO NPs can be effectively separated into ZnO particles and Zn ions: (1) ZnO NPs were recovered from the precipitated TX-114-rich phase after CPE as intact particles (Figure 2); (2) the hydrodynamic radii and primary particle sizes of pristine ZnO NPs obtained by CPE were statistically similar to those of pristine (Table 1, Figure 2), suggesting that particle size and size distribution were not affected during CPE procedure; (3) more than 82% of pristine ZnO NPs under all variable CPE conditions and ~93% of ZnO NPs in HA at 45 °C were recovered in the precipitates after CPE (Figure 2C), suggesting the efficacy of CPE for separating ZnO NPs as particle form; and (4) small amounts (less than 2%) of ZnO NPs were detected in supernatants as Zn ions (Figure 2C), indicating that only a minimum amount of Zn ions was released from ZnO NPs during CPE. This was also confirmed by low dissolution property (less than 2%) of ZnO NPs in different dispersants (Figure 3A). On the other hand, zeta potential values were affected and changed to slightly negative charges, approaching zero zeta potentials in all cases after CPE (Table 1). It is known that zeta potentials close to zero are optimal for the formation of NPs-TX-114 micelles [30,31]. The results obtained with pristine ZnO NPs suggest that TX-114-based CPE, especially in HA as a dispersant at 45 °C, can effectively separate ZnO particles and Zn ions without affecting primary particle size, size distribution, or morphology.

The dissolution property of ZnO NPs was highly affected by food matrices and cell culture medium (Figure 3B,C). The solubilities of ZnO NPs in coffee mix, skim milk, and milk ranged from ~30–49% (Figure 3B), although the pHs of ZnO-NP-spiked food matrices were close to neutral, except for sports drink (pH 3.3). It is known that ZnO can be dissolved into Zn ions under acidic conditions [39,40,41]. Thus, the high solubility (~91%) of ZnO in sports drink can be explained by the low pH of the sports drink. Increased solubilities of ZnO in other food matrices, such as coffee mix, skim milk, and milk, seem to be related to its interaction with food components. Meanwhile, the solubilities of ZnO NPs in MEM cell culture medium (pH 7.0) were ~18% to 25% over the incubation time (Figure 3C), which likely resulted from their interactions with various components found in the MEM [38,42]. The solubility of ZnO could, therefore, be highly affected by the presence of food or biomatrices.

The efficacy of CPE was also confirmed using ZnO-NP-spiked foods, showing the reliability and accuracy of the CPE for fate determination of ZnO NPs in foods: (1) DLS results recovered from the precipitates of ZnO-NP-spiked coffee mix, skim milk, and milk after CPE had similar hydrodynamic radii compared to pristine ZnO NPs (Supplementary Figure S1), indicating that ZnO NPs were well recovered as intact particle forms after CPE; (2) no particle forms were detected in the precipitates of ZnO-NP-spiked sports drink after CPE (Supplementary Figure S1), suggesting the complete dissolution of ZnO into Zn ions; (3) SEM-EDS analysis revealed the presence of Zn elements recovered from the precipitates of all ZnO-NP-spiked foods after CPE, except in sports drink (Figure 4A,B), which was in good agreement with DLS results; and (4) ~51–60% of ZnO were present as particles in coffee mix, skim milk, and milk, whereas almost all ZnO NPs were detected as Zn ions in sports drink after CPE, which is highly consistent with the dissolution properties of ZnO (Figure 3B). Indeed, no significant differences were found between solubilized Zn ions in food matrices (Figure 3B) and Zn ions recovered after CPE (Figure 4C, *p* > 0.05). Hence, the fate of ZnO NPs in food matrices can be determined as both intact particle and Zn ion forms by applying CPE, without affecting particle size and solubility. The total Zn recoveries (%) from both ZnO particles and Zn ions ranged from 92.6% to 97.5%, suggesting the accuracy of the analytical procedure.

The same trends were observed in commercial foods in which ZnO addition was indicated on product labeling. Most ZnO particles were present as Zn ions in liquid foods (functional peptide beverage and fruit juice), while the fate of ZnO was found to be mainly the particle forms in powdered foods (chocolate powder, powdered probiotics, and cereals, Figure 5). It is worth noting that another functional mineral beverage fortified with Zn gluconate was found to contain Zn ions after CPE (Figure 5F), supporting the reliability of the results. The pHs of liquid beverages were 2.7–4.7, which affected ZnO dissolution property due to the fact that ZnO dissolves more rapidly in acidic solutions. The slight presence of ZnO as particles in liquid beverages seems to be attributable to a Zn ion complex formed with other food components. The total Zn recoveries (%) from both ZnO and Zn ions ranged from 96.9% to 102.8%, and was calculated based on composition labeling in commercial foods and ICP-AES analysis, supporting the accuracy of the CPE procedure. Zn ion ratios in ZnO-NP-spiked powdered foods (Figure 4) were higher than those in commercial powdered or dried foods (Figure 5). The various processing steps used for commercial food products, such as formulation, mixing, and thermal or drying treatment, may increase the stability of ZnO in processed, powdered foods, contributing to its low dissolution in food matrices. Taken together, it is probable that ZnO as a food additive is primarily present as a particle in powdered or dried foods, but can be easily decomposed into Zn ions in liquid foods.

The intracellular fate of ZnO NPs, determined by CPE followed by ICP-AES analysis, revealed that ZnO NPs were taken up in particle forms, but slowly dissolved into Zn ions after a certain time inside cells (Figure 6A). A portion of dissolved Zn ions from ZnO in cell culture medium can be also rapidly taken up by cells, considering that the dissolution property of ZnO NPs in MEM is ~18–25% (Figure 3C). Vandebriel et al. also demonstrated that ZnO NPs can be taken up by cells by in both particle and ionic forms, which is consistent with our findings [43]. Paek et al. reported that Zn ions can be more rapidly and massively absorbed into the bloodstream after oral administration in rats [18], which may support the rapid cellular uptake of Zn ions compared to ZnO (Figure 6A). Taken together, both ZnO NPs and Zn ions can be internalized into cells, but the major fate of ZnO NPs is to become ionized inside cells over time. Gilbert et al. demonstrated that ZnO NPs were completely dissolved into Zn ions after cellular internalization [12]. Wang et al. also reported that ZnO NPs were internalized into cells by endocytosis and localized within acidic lysosomes, releasing Zn ions from internalized ZnO NPs [44,45,46]. ZnO NPs were reported to cause cytotoxicity associated with an increase in the Zn ions released inside cells [47,48]. However, contradictory results were obtained by confocal microscopy using a Zn–selective fluorescent probe, showing elevated Zn ion levels at 0.5–1 h and decreased Zn ions thereafter (Figure 6B). The discrepancy might be explained by complex formation between Zn ions and other molecular ligands inside cells, which was evidenced by Zn–S bond formation in tissues after oral administration of ZnO NPs in rats [15]. It is, therefore, strongly likely that ZnO NPs are dissolved into Zn ions and form Zn–molecular ligand complexes after internalization into cells, which is in good agreement with the results obtained by Gilbert et al. [12].

The intestinal transport and absorption fate of ZnO NPs, evaluated using in vitro models of human intestinal barriers and ex vivo everted small intestine sacs, was determined to be primarily Zn ion forms. This result suggests that ZnO NPs can be taken up by cells in both particle and ionized forms (Figure 6A), but most ZnO particles are dissolved into Zn ions during intestinal transit and absorption (Figure 7). Our previous report demonstrated that the ex vivo solubility of ZnO NPs in rat-extracted intestinal fluid was ~9% [23], supporting their high dissolution during intestinal transit. Thus, the major fate of absorbed ZnO NPs in the small intestine is likely to be the ionized forms. However, a small amount of ZnO can be also transported by M cells and absorbed as particle form (Figure 7B,C), suggesting different toxicokinetic behaviors of ZnO compared to those of Zn ions, as reported by previous research [18]. Intestinal transport of NPs by M cells was also demonstrated [23,49,50].

## 4. Materials and Methods

### 4.1. Materials

ZnO NPs (<100 nm), D-(+)-glucose, humic acid (sodium salt), TX-114, EDTA, formalin, ammonium chloride (NH_4_Cl), and monosodium phosphate (NaH_2_PO_4_) were purchased from Sigma-Aldrich (St. Louis, MO, USA). Sodium hydroxide (NaOH), sodium chloride (NaCl), potassium chloride (KCl), calcium chloride (CaCl_2_), magnesium chloride (MgCl_2_), sodium bicarbonate (NaHCO_3_), nitric acid (HNO_3_), and hydrogen peroxide (H_2_O_2_) were supplied by Samchun Pure Chemical Co., Ltd. (Pyeongtaek, Gyeonggi-do, Korea). Conical-bottom glass centrifuge tubes (15 mL) were obtained from Daeyoung Science (Seoul, Korea). MEM, Roswell Park Memorial Institute (RPMI) 1640 medium, Dulbecco’s modified eagle’s medium (DMEM), heat-inactivated fetal bovine serum (FBS), penicillin, streptomycin, Dulbecco’s phosphate-buffered saline (DPBS), and phosphate-buffered saline (PBS) were purchased from Welgene Inc. (Gyeongsan, Gyeongsangbuk-do, Korea). N-(6-Methoxy-8-quinolyl)-p-toluenesulfonamide (TSQ) was obtained from Enzo Life Science Inc. (Farmingdale, NY, USA). Matrigel^®^ was from Corning Inc. (Corning, NY, USA). Transwell^®^ polycarbonate inserts were purchased from SPL Life Science Co., Ltd. (Pocheon, Gyeonggi-do, Korea).

Commercial foods used for the spiking experiment were as follows: coffee mix, skim milk, milk, and sports drink. Commercial Zn-fortified products analyzed were as follows: chocolate powder (ZnO added), powdered probiotics (ZnO added), cereals (ZnO added), functional peptide beverage (ZnO added), fruit juice (ZnO added), and functional mineral beverage (Zn gluconate added), all from international brands found in markets in Seoul, Republic of Korea, in 2019.

### 4.2. Characterization

Primary particle size, shape, and chemical characterization were determined by FE-SEM (JSM-7100F, JEOL, Tokyo, Japan), equipped with EDS (Aztec, Oxford Instruments, Abingdon, UK). Zeta potentials and hydrodynamic radii of particles (1 mg/mL) were measured with Zetasizer Nano System (Malvern Instruments, Worcestershire, UK) after stirring for 30 min, sonication for 15 min (Bransonic 5800, Branson Ultrasonics, Danbury, CT, USA), and dilution (0.1 mg/mL).

### 4.3. Optimization of CPE for Pristine ZnO NPs

ZnO NPs (100 μg/mL) were dispersed in 7 mL of DDW, HA (final concentration of 10 μg/mL), or Glc (final concentration of 1% (*w*/*v*)) solution by stirring for 30 min, followed by sonication for 15 min. CPE was processed as described by Majedi et al. [33]. The suspensions (7 mL) were transferred to conical-bottom glass centrifuge tubes and the pH was adjusted to 10 by adding an NaOH solution. Next, 5% (*w*/*v*) TX-114 (0.5 mL) and 0.2 M NaCl solution (0.75 mL) were added to the suspensions. After dilution to 10 mL with DDW, the mixtures were incubated for 30 min at 37 or 45 °C and centrifuged for 5 min at 2500× *g* at 25 °C. The precipitates and supernatants were analyzed by ICP-AES (JY2000 Ultrace, HORIBA Jobin Yvon, Longjumeau, France) after digestion with HNO_3_ as described in Section 4.12.

### 4.4. Fate Determination of ZnO NPs in Food Matrices

ZnO NPs (10 mg) were spiked into 10 g of powdered foods, such as coffee mix and skim milk powder, and mixed well. Next, 0.1 g of the mixed powders were dispersed in 7 mL of HA solution, and the dispersions were stirred for 30 min followed by sonication for 15 min. In parallel, 10 mg of ZnO NPs were spiked with 100 mL of HA-added liquid foods, such as milk and sports drink. Spiked samples (1 mL) were then diluted to 7 mL by adding HA solution and stirring for 30 min, followed by sonication for 15 min. The Zn concentrations were chosen based on the ADI for Zn in Zn-fortified functional foods (2.55–12 mg) in the Republic of Korea [8]. The same procedure was applied as described in Section 4.3.

### 4.5. Dissolution Property of ZnO NPs in Food/Bio Matrices

ZnO NPs in DDW, different dispersants, or food matrices were prepared and stirred for 30 min. The same concentration of ZnO NPs for optimization of CPE and fate determination in food matrices were used. ZnO NPs (50 μg/mL) in cell culture medium MEM were incubated for 0.5, 1, 6, and 24 h with gentle shaking (180 rpm) at 37 °C. The ZnO suspensions were then centrifuged (16,000× *g*) for 15 min, and the supernatants were subjected to ICP-AES analysis after pre-digestion as described in Section 4.12.

### 4.6. Fate Determination of ZnO in Zn-Fortified Commercial Foods

Zn-fortified powdered foods (10 g) were homogenized in an agate mortar. Samples thus homogenized (0.1 g) were dispersed in 7 mL of HA solution, and the dispersions were stirred for 30 min and sonicated for 15 min. Commercial Zn-fortified beverages (1 mL) were diluted to 7 mL by adding HA solution, and the solutions were stirred for 30 min followed by sonication for 15 min. The same procedure was applied as described in Section 4.3.

### 4.7. Cell Culture

Human intestinal epithelial Caco-2 cells were purchased from the Korean Cell Line Bank (KCLB; Seoul, Korea). The cells were cultured in MEM containing 10% FBS, 100 units/mL of penicillin, and 100 µg/mL of streptomycin in a 5% CO_2_ incubator at 37 °C.

### 4.8. Cellular Uptake and Intracellular Fate of ZnO NPs

The cells were plated at a density of 1 × 10^6^ cells/well and incubated with ZnO NPs (50 μg/mL) for 0.5, 1, 6, and 24 h. After washing three times with DPBS, 5 mM EDTA in DPBS was used to treated the cells for 40 s in order to remove adsorbed NPs on the surface of cell membrane. After washing with PBS three times, the cells were harvested with a scraper, centrifuged, and re-suspended in 1 mL of DDW to determine the intracellular fate of ZnO NPs by CPE and cellular uptake quantification by ICP-AES. Cells in the absence of ZnO NPs were used as controls.

The suspended cells (1 mL) were transferred to conical-bottom glass centrifuge tubes and sonicated four times for 10 s on ice with a 150 W ultrasonic processor (Sonics & Materials Inc., Newtown, CT, USA). After dilution to 7 mL by adding HA solution, the same procedure was applied as described in Section 4.3.

### 4.9. Intracellular Fate of ZnO NPs by Confocal Microscopy

The cells were plated at a density of 2 × 10^4^ cells on a glass coverslip, and ZnO NPs (50 μg/mL) were treated for 0.5, 1, 6, and 24 h. After washing with DPBS, the cells were fixed with 500 μL of freshly made 3.7% formalin (containing 1.5% methanol) in DPBS on ice for 20 min. Next, 50 mM NH_4_Cl in DPBS was added and incubation was continued on ice for 30 min. After washing twice with DPBS, 50 μL of 30 μM fluorescent probe for Zn ions, TSQ, was added and incubated for 30 min in the dark at room temperature. Finally, the cells were rinsed three times with DPBS and visualized using a D-Eclipse C1 confocal microscope (Nikon Instech. Co., Kawasaki, Japan), equipped with Ar (488 nm) and HeNe (543 nm) lasers. Image acquisition and analysis were performed with EZ-C1 2.3 software (Nikon Instech. Co., Kawasaki, Japan). Each experiment was repeated twice on separate days.

### 4.10. Intestinal Transport Fate of ZnO NPs

Human Burkitt’s lymphoma Raji B cells, supplied from the KCLB, were cultured in RPMI 1640 medium containing FBS (10%), non-essential amino acids (1%), L-glutamine (1%), penicillin (100 units/mL), and streptomycin (100 μg/mL) in a 5% CO_2_ incubator at 37 °C. The FAE model, mimicking M cells, was established as described previously [23,51]. After coating Transwell^®^ polycarbonate inserts with Matrigel^®^ matrix prepared in serum-free DMEM for 2 h, supernatants were removed, and inserts were then washed with serum-free DMEM. Caco-2 cells (1 × 10^6^ cells/well) were seeded on the apical sides and grown for 14 days. Lymphoma Raji B cells (1 × 10^6^ cells/well) were added to the basolateral sides, and these co-cultures were maintained for 5 days until trans epithelial electrical resistance (TEER) values reached 150–200 Ω cm^2^. The apical medium of the monolayers was then replaced by medium containing ZnO NPs (50 μg/mL), and incubation continued for 0.5 and 6 h.

Caco-2 monoculture was also used to evaluate the transported fate of ZnO NPs through intestinal epithelial tight junction barrier. Caco-2 cells (4.5 × 10^5^ cells/well) were seeded on upper inserts and further cultured for 21 days (TEER ≥ 300 Ω cm^2^). Apical medium of the monolayers was then replaced by medium containing ZnO NPs (50 μg/mL), and incubation continued for 0.5 and 6 h.

Basolateral solutions (1 mL) were collected in a conical-bottom glass centrifuge tube, and diluted to 7 mL with HA solution. The same procedure was applied as described in Section 4.3.

### 4.11. Intestinal Absorption Fate of ZnO NPs

Eight-week-old male Sprague Dawley (SD; 200–250 g) rats were purchased from Koatech Co. (Pyeongtaek, Gyeonggi-do, Korea). Animals were housed in plastic laboratory animal cages in a ventilated room, maintained at 20 ± 2 °C and 60% ± 10% relative humidity with a 12 h light/dark cycle. Water and commercial complete laboratory food for rats were available ad libitum. Animals were environmentally acclimated for 5 days before treatment. All animal experiments were performed in compliance with the guideline issued by the Animal and Ethics Review Committee of Seoul Women’s University (SWU IACUC-2019A-1), Republic of Korea.

Everted small intestine sacs were prepared as previously described [52]. Briefly, two male rats were fasted overnight (water available) and sacrificed by CO_2_ euthanasia. The small intestines were collected, washed three times with Tyrode’s solution (containing 0.8 g of NaCl, 0.02 g of KCl, 0.02 g of CaCl_2_, 0.01 g of MgCl_2_, 0.1 g of NaHCO_3_, 0.005 g of NaH_2_PO_4_, and 0.1 g of glucose in 100 mL of distilled water), cut into sections (5 cm in length), and everted on a puncture needle (0.8 mm in diameter). After one end was clamped, the everted sacs were filled with 200 µL of Tyrode’s solution and then tied using silk braided sutures. Each sac was placed in a six well plate containing 3 mL of ZnO NPs (50 μg/mL) for 0.5 and 6 h in a humidified 5% CO_2_ atmosphere at 37 °C. The solution in the interior sac was collected and the same procedure was applied after dilution to 7 mL with HA solution, as described in Section 4.3.

### 4.12. ICP-AES Analysis

All samples were pre-digested with 10 mL of ultrapure HNO_3_ at ~160 °C, and 1 mL of H_2_O_2_ solution was added and heated until the samples were colorless and until the solution was completely evaporated. The digested samples were diluted with 3 mL of DDW, and total Zn concentrations were determined by ICP-AES (JY2000 Ultrace, HORIBA Jobin Yvon).

### 4.13. Statistical Analysis

Results are expressed as means ± standard deviations. One-way analysis of variance with Tukey’s test in SAS Ver.9.4 (SAS Institute Inc., Cary, NC, USA) was used to determine the significances of intergroup differences. Statistical significance was accepted for *p* values < 0.05.

## 5. Conclusions

Surfactant TX-114-based CPE was optimized and established for fate determination of ZnO in commercial foods and human intestinal cells. The solubility of ZnO was not affected by dispersants used for CPE, but was highly affected by food matrices or cell culture medium, showing dissolved fate to some extent. ZnO was found to be mainly present as particle forms in powdered or dried foods, whereas its major fate in liquid beverages was in Zn ionic form. On the other hand, ZnO NPs were internalized into cells as both particles and Zn ions, but slowly dissolved into Zn ions upon time, probably forming Zn–ligand complexes inside the cells. ZnO NPs were found to be transported through intestinal barriers and absorbed in the small intestine primarily as Zn ions. However, a portion of ZnO NPs could be absorbed into the body as particles. The toxicity of ZnO NPs is, therefore, likely to be mainly associated with Zn ion toxicity, but long-term potential toxicity resulting from particle forms cannot be completely excluded. These findings will be useful for understanding the potential toxicity of ZnO NPs and for their wide application to commercial foods at safe levels.

## Figures and Tables

**Table 1:** Hydrodynamic radii and zeta potentials of pristine ZnO nanoparticles (NPs) in different CPE conditions.

Dispersant Type	Incubation Temperature (°C)	Z-Average Size (nm)			Zeta Potential (mV)		
		Step 1	Step 2	Step 3	Step 1	Step 2	Step 3
ZnO in DDW	37	261.27 ± 3.59 ^A,a^	1716.33 ± 93.38 ^A,c^	739.57 ± 113.49 ^B,b^	25.9 ± 0.99 ^A,a^	−0.98 ± 0.05 ^B,b^	−1.62 ± 0.14 ^A,b^
	45	261.27 ± 3.59 ^A,a^	1717.00 ± 120.68 ^A,c^	857.97 ± 56.61 ^BC,b^			
ZnO in HA	37	260.43 ± 4.22 ^A,a^	1653.67 ± 123.52 ^A,b^	345.67 ± 17.05 ^A,a^	−34.63 ± 1.33 ^C,c^	−0.77 ± 0.02 ^A,a^	−4.92 ± 0.44 ^B,b^
	45	260.43 ± 4.22 ^A,a^	2272.33 ± 279.40 ^B,b^	338.90 ± 10.91 ^A,a^			
ZnO in Glc	37	777.57 ± 19.03 ^B,a^	1466.33 ± 22.72 ^A,c^	897.13 ± 35.72 ^B^^,^^C,b^	16.63 ± 0.42 ^B,a^	−1.37 ± 0.10 ^C,b^	−1.68 ± 0.17 ^A,b^
	45	777.57 ± 19.03 ^B,a^	1685.67 ± 147.97 ^A,b^	1073.53 ± 171.95 ^C,a^			

Different upper-case letters (A,B,C) indicate significant differences among different CPE conditions (*p* < 0.05). Different lower-case letters (a,b,c) indicate significant differences among different CPE steps (*p* < 0.05). Abbreviation: DDW, distilled and deionized water; HA, humic acid; Glc, glucose.

## Supplementary Materials

Supplementary materials can be found at https://www.mdpi.com/1422-0067/21/2/433/s1. Figure S1: Hydrodynamic radii of pristine ZnO and ZnO obtained by CPE in ZnO-NP-spiked foods.

## References

[1] Peters R., Kramer E., Oomen A.G., Rivera Z.E., Oegema G., Tromp P.C., Fokkink R., Rietveld A., Marvin H.J., Weigel S. (2012). Presence of nano-sized silica during in vitro digestion of foods containing silica as a food additive. ACS Nano.

[2] Skocaj M., Filipic M., Petkovic J., Novak S. (2011). Titanium dioxide in our everyday life; is it safe?. Radiol. Oncol..

[3] Swain P.S., Rao S.B.N., Rajendran D., Dominic G., Selvaraju S. (2016). Nano zinc, an alternative to conventional zinc as animal feed supplement: A review. Anim. Nutr..

[4] Bonaventura P., Benedetti G., Albarede F., Miossec P. (2015). Zinc and its role in immunity and inflammation. Autoimmun. Rev..

[5] Hirano T., Murakami M., Fukada T., Nishida K., Yamasaki S., Suzuki T. (2008). Roles of zinc and zinc signaling in immunity: Zinc as an intracellular signaling molecule. Adv. Immunol..

[6] MacDonald R.S. (2000). The role of zinc in growth and cell proliferation. J. Nutr..

[7] U.S. Food and Drug Administration (FDA) GRAS Notice. https://www.accessdata.fda.gov/scripts/cdrh/cfdocs/cfCFR/CFRSearch.cfm?fr=182.8991.

[8] Republic of Korea Ministry of Food and Drug Safety (MFDS) Criteria and Standard of Health Functional Foods. https://www.mfds.go.kr/brd/m_211/view.do?seq=14375.

[9] FDA Food Additive Status List. https://www.fda.gov/food/food-additives-petitions/food-additive-status-list#ftnZ.

[10] Kononenko V., Repar N., Marusic N., Drasler B., Romih T., Hocevar S., Drobne D. (2017). Comparative in vitro genotoxicity study of ZnO nanoparticles, ZnO macroparticles and ZnCl_2_ to MDCK kidney cells: Size matters. Toxicol. In Vitro.

[11] Liu J., Kang Y., Yin S., Song B., Wei L., Chen L., Shao L. (2017). Zinc oxide nanoparticles induce toxic responses in human neuroblastoma SHSY5Y cells in a size-dependent manner. Int. J. Nanomed..

[12] Gilbert B., Fakra S.C., Xia T., Pokhrel S., Madler L., Nel A.E. (2012). The fate of ZnO nanoparticles administered to human bronchial epithelial cells. ACS Nano.

[13] Majedi S.M., Lee H.K., Kelly B.C. (2013). Role of water temperature in the fate and transport of zinc oxide nanoparticles in aquatic environment. J. Phys. Conf. Ser..

[14] Sivry Y., Gelabert A., Cordier L., Ferrari R., Lazar H., Juillot F., Menguy N., Benedetti M.F. (2014). Behavior and fate of industrial zinc oxide nanoparticles in a carbonate-rich river water. Chemosphere.

[15] Baek M., Chung H.E., Yu J., Lee J.A., Kim T.H., Oh J.M., Lee W.J., Paek S.M., Lee J.K., Jeong J. (2012). Pharmacokinetics, tissue distribution, and excretion of zinc oxide nanoparticles. Int. J. Nanomed..

[16] Cho W.S., Kang B.C., Lee J.K., Jeong J., Che J.H., Seok S.H. (2013). Comparative absorption, distribution, and excretion of titanium dioxide and zinc oxide nanoparticles after repeated oral administration. Part. Fibre Toxicol..

[17] Seok S.H., Cho W.S., Park J.S., Na Y., Jang A., Kim H., Cho Y., Kim T., You J.R., Ko S. (2013). Rat pancreatitis produced by 13-week administration of zinc oxide nanoparticles: Biopersistence of nanoparticles and possible solutions. J. Appl. Toxicol..

[18] Paek H.J., Lee Y.J., Chung H.E., Yoo N.H., Lee J.A., Kim M.K., Lee J.K., Jeong J., Choi S.J. (2013). Modulation of the pharmacokinetics of zinc oxide nanoparticles and their fates in vivo. Nanoscale.

[19] Wang B., Feng W., Wang M., Wang T., Gu Y., Zhu M., Ouyang H., Shi J., Zhang F., Zhao Y. (2008). Acute toxicological impact of nano- and submicro-scaled zinc oxide powder on healthy adult mice. J. Nanopart. Res..

[20] Avramescu M.L., Rasmussen P.E., Chenier M., Gardner H.D. (2017). Influence of pH, particle size and crystal form on dissolution behaviour of engineered nanomaterials. Environ. Sci. Pollut. Res..

[21] Cho W.S., Duffin R., Howie S.E., Scotton C.J., Wallace W.A., Macnee W., Bradley M., Megson I.L., Donaldson K. (2011). Progressive severe lung injury by zinc oxide nanoparticles; the role of Zn^2+^ dissolution inside lysosomes. Part. Fibre Toxicol..

[22] Liu J.H., Ma X., Xu Y., Tang H., Yang S.T., Yang Y.F., Kang D.D., Wang H., Liu Y. (2017). Low toxicity and accumulation of zinc oxide nanoparticles in mice after 270-day consecutive dietary supplementation. Toxicol. Res..

[23] Yu J., Kim H.J., Go M.R., Bae S.H., Choi S.J. (2017). ZnO interactions with biomatrices: Effect of particle size on ZnO-protein corona. Nanomaterials.

[24] Bae S.H., Yu J., Lee T.G., Choi S.J. (2018). Protein food matrix-ZnO nanoparticle interactions affect protein conformation, but may not be biological responses. Int. J. Mol. Sci..

[25] Go M.R., Yu J., Bae S.H., Kim H.J., Choi S.J. (2018). Effects of interactions between ZnO nanoparticles and saccharides on biological responses. Int. J. Mol. Sci..

[26] Jo M.R., Chung H.E., Kim H.J., Bae S.H., Go M.R., Yu J., Choi S.J. (2016). Effects of zinc oxide nanoparticle dispersants on cytotoxicity and cellular uptake. Mol. Cell Toxicol..

[27] Zukiene R., Snitka V. (2015). Zinc oxide nanoparticle and bovine serum albumin interaction and nanoparticles influence on cytotoxicity in vitro. Colloids Surf. B.

[28] Chao J.B., Liu J.F., Yu S.J., Feng Y.D., Tan Z.Q., Liu R., Yin Y.G. (2011). Speciation analysis of silver nanoparticles and silver ions in antibacterial products and environmental waters via cloud point extraction-based separation. Anal. Chem..

[29] Hartmann G., Schuster M. (2013). Species selective preconcentration and quantification of gold nanoparticles using cloud point extraction and electrothermal atomic absorption spectrometry. Anal. Chim. Acta.

[30] Majedi S.M., Kelly B.C., Lee H.K. (2014). Evaluation of a cloud point extraction approach for the preconcentration and quantification of trace CuO nanoparticles in environmental waters. Anal. Chim. Acta.

[31] Liu J.F., Chao J.B., Liu R., Tan Z.Q., Yin Y.G., Wu Y., Jiang G.B. (2009). Cloud point extraction as an advantageous preconcentration approach for analysis of trace silver nanoparticles in environmental waters. Anal. Chem..

[32] Yu S.J., Chao J.B., Sun J., Yin Y.G., Liu J.F., Jiang G.B. (2013). Quantification of the uptake of silver nanoparticles and ions to HepG2 cells. Environ. Sci. Technol..

[33] Majedi S.M., Lee H.K., Kelly B.C. (2012). Chemometric analytical approach for the cloud point extraction and inductively coupled plasma mass spectrometric determination of zinc oxide nanoparticles in water samples. Anal. Chem..

[34] Azizi N.A.M., Rahim N.Y., Raoov M., Asman S. (2019). Optimisation and evaluation of zinc in food samples by cloud point extraction and spectrophotometric detection. Sci. Res. J..

[35] Tabrizi A.B. (2007). Cloud point extraction and spectrofluorimetric determination of aluminium and zinc in foodstuffs and water samples. Food Chem..

[36] Park K., Lee Y. (2013). The stability of citrate-capped silver nanoparticles in isotonic glucose solution for intravenous injection. J. Toxicol. Environ. Health A.

[37] Hamilton R.F., Buckingham S., Holian A. (2014). The effect of size on Ag nanosphere toxicity in macrophage cell models and lung epithelial cell lines is dependent on particle dissolution. Int. J. Mol. Sci..

[38] Reed R.B., Ladner D.A., Higgins C.P., Westerhoff P., Ranville J.F. (2012). Solubility of nano-zinc oxide in environmentally and biologically important matrices. Environ. Toxicol. Chem..

[39] Adamcakova-Dodd A., Stebounova L.V., Kim J.S., Vorrink S.U., Ault A.P., O’Shaughnessy P.T., Grassian V.H., Thorne P.S. (2014). Toxicity assessment of zinc oxide nanoparticles using sub-acute and sub-chronic murine inhalation models. Part. Fibre Toxicol..

[40] Bian S.W., Mudunkotuwa I.A., Rupasinghe T., Grassian V.H. (2011). Aggregation and dissolution of 4 nm ZnO nanoparticles in aqueous environments: Influence of pH, ionic strength, size, and adsorption of humic acid. Langmuir.

[41] Chaúque E.F.C., Zvimba J.N., Ngila J.C., Musee N. (2014). Stability studies of commercial ZnO engineered nanoparticles in domestic wastewater. Phys. Chem. Earth.

[42] Xia T., Kovochich M., Liong M., Madler L., Gilbert B., Shi H., Yeh J.I., Zink J.I., Nel A.E. (2008). Comparison of the mechanism of toxicity of zinc oxide and cerium oxide nanoparticles based on dissolution and oxidative stress properties. ACS Nano.

[43] Vandebriel R.J., De Jong W.H. (2012). A review of mammalian toxicity of ZnO nanoparticles. Nanotechnol. Sci. Appl..

[44] Wang B., Zhang Y., Mao Z., Yu D., Gao C. (2014). Toxicity of ZnO nanoparticles to macrophages due to cell uptake and intracellular release of zinc ions. J. Nanosci. Nanotechnol..

[45] Condello M., De Berardis B., Ammendolia M.G., Barone F., Condello G., Degan P., Meschini S. (2016). ZnO nanoparticle tracking from uptake to genotoxic damage in human colon carcinoma cells. Toxicol. In Vitro.

[46] Roy R., Parashar V., Chauhan L.K., Shanker R., Das M., Tripathi A., Dwivedi P.D. (2014). Mechanism of uptake of ZnO nanoparticles and inflammatory responses in macrophages require PI3K mediated MAPKs signaling. Toxicol. In Vitro.

[47] Shen C., James S.A., de Jonge M.D., Turney T.W., Wright P.F., Feltis B.N. (2013). Relating cytotoxicity, zinc ions, and reactive oxygen in ZnO nanoparticle-exposed human immune cells. Toxicol. Sci..

[48] Shi L.E., Li Z.H., Zheng W., Zhao Y.F., Jin Y.F., Tang Z.X. (2014). Synthesis, antibacterial activity, antibacterial mechanism and food applications of ZnO nanoparticles: A review. Food Addit. Contam. A.

[49] Fievez V., Plapied L., Plaideau C., Legendre D., des Rieux A., Pourcelle V., Freichels H., Jerome C., Marchand J., Preat V. (2010). In vitro identification of targeting ligands of human M cells by phage display. Int. J. Pharm..

[50] Gamboa J.M., Leong K.W. (2013). In vitro and in vivo models for the study of oral delivery of nanoparticles. Adv. Drug Deliv. Rev..

[51] Des Rieux A., Fievez V., Theate I., Mast J., Preat V., Schneider Y.J. (2007). An improved in vitro model of human intestinal follicle-associated epithelium to study nanoparticle transport by M cells. Eur. J. Pharm. Sci..

[52] Gu T., Yao C., Zhang K., Li C., Ding L., Huang Y., Wu M., Wang Y. (2018). Toxic effects of zinc oxide nanoparticles combined with vitamin C and casein phosphopeptides on gastric epithelium cells and the intestinal absorption of mice. RSC Adv..

