# Supplementary material for: Fate Determination of ZnO in Commercial Foods and Human Intestinal Cells
Source: Int J Mol Sci. 2020 Jan 9;21(2):433. doi: 10.3390/ijms21020433 (PMC7014048; doi:10.3390/ijms21020433)
Supplement: Supplementary file 1 [file ijms-21-00433-s001.pdf]

Fate Determination of ZnO in Commercial Foods and Human Intestinal Cells

Ye-Rin Jeon, Jin Yu, Soo-Jin Choi*

Division of Applied Food System, Major of Food Science & Technology, Seoul Women's University, Seoul 01797, Republic of Korea; yrjeon0715@swu.ac.kr (Y.-R.J.); ky5031@swu.ac.kr (J.Y.)

* Correspondence: sjchoi@swu.ac.kr; Tel.: +82-2-970-5634; Fax: +82-2-970-5977

Received: date; Accepted: date; Published: date

Supplementary Materials

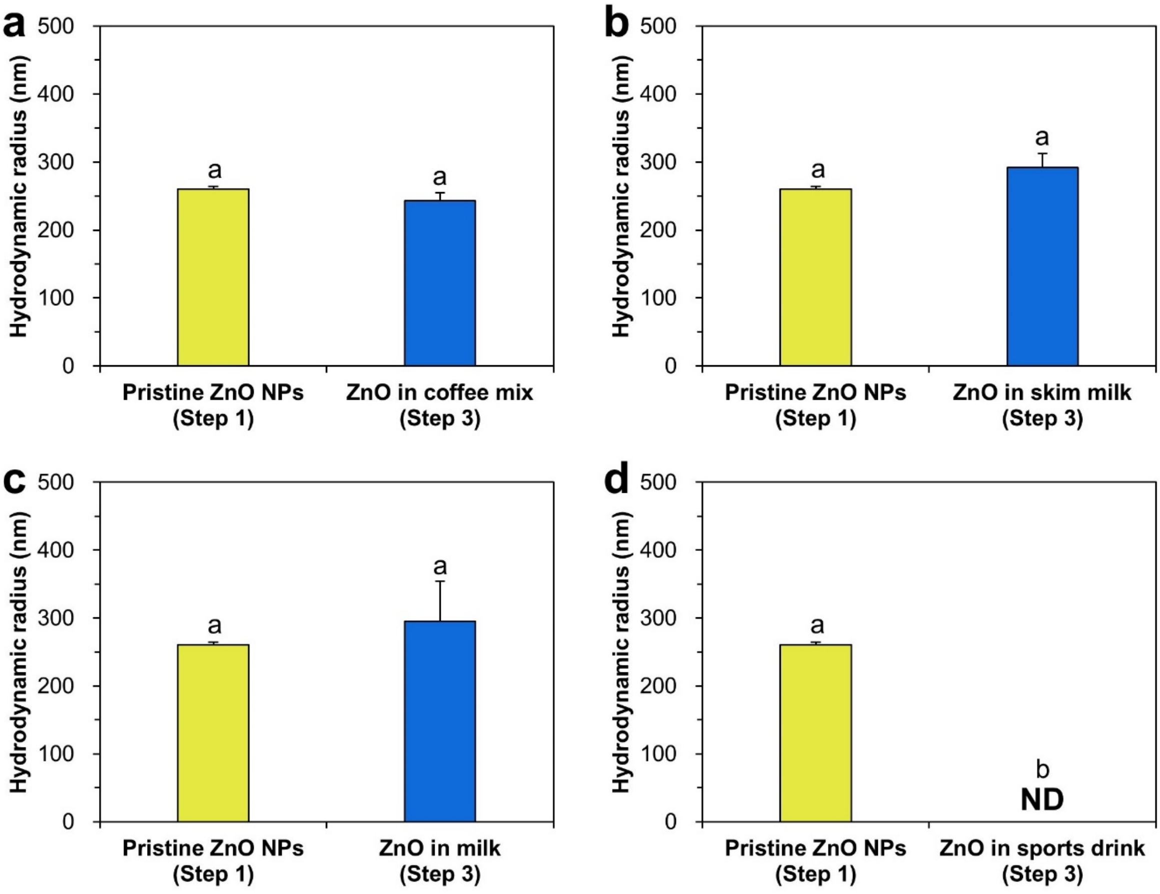

Figure S1. Hydrodynamic radii of pristine ZnO and ZnO obtained by CPE in ZnO NPs-spiked (a) coffee mix, (b) skim milk, (c) milk, and (d) sports drink. Different lower case letters (a,b) indicate significant differences between pristine ZnO NPs before CPE (step 1) and after ZnO NPs (step 3) ($P < 0.05$). ND: Not detectable.
